# Supplementary material for: Impact of Toceranib Phosphate and Carprofen on Survival and Quality of Life in Dogs with Inflammatory Mammary Carcinomas
Source: Vet Sci. 2024 Sep 13;11(9):430. doi: 10.3390/vetsci11090430 (PMC11435629; doi:10.3390/vetsci11090430)
Supplement: Supplementary file 1 [file vetsci-11-00430-s001.zip › vetsci-3161580-supplementary.pdf]

QUALITY OF LIFE QUESTIONNAIRE FOR CANINE CANCER TREATMENT

DATE OF THE QUESTIONNAIRE: ___________ OWNER_______________________________________

NAME OF THE PERSON COMPLETING THE QUESTIONNAIRE:___________________________

NAME OF THE PATIENT___________________

INSTRUCTIONS: Indicate your opinion about your pet's health by circling the numbers on the scale next to each question.

EXAMPLE:	1	2	3	4	5
	Disagree		Neutral		Agree

**HAPINNESS**					
My dog wants to play	1	2	3	4	5
My dog responds to my presence	1	2	3	4	5
My dog enjoys life	1	2	3	4	5
**MENTAL STATUS**					
My dog has more good days than bad days	1	2	3	4	5
My dog sleeps more, is awake less	1	2	3	4	5
My dog seems dull or depressed, not alert	1	2	3	4	5
**PAIN**					
My dogs is in pain	1	2	3	4	5
My dog pants frequently, even at rest	1	2	3	4	5
My dogs shakes or trembles occasionally	1	2	3	4	5
**APPETITE**					
My dog eats the usual amount of food	1	2	3	4	5
My dog acts nauseous or vomits	1	2	3	4	5
My dog eats treats/snacks	1	2	3	4	5
**HYGIENE**					
My dog keeps him/herself clean	1	2	3	4	5
My dog smells like urine or has skin irritation	1	2	3	4	5
My dog´s hair is greasy, matted, rough looking	1	2	3	4	5
**WATER INTAKE (HYDRATION)**					
My dog drinks adequately	1	2	3	4	5
My dog has diarrhea	1	2	3	4	5
Mi do gis urinating a normal amount	1	2	3	4	5
**MOBILITY**					
My dog moves normally	1	2	3	4	5
My dog lays in one place all day long	1	2	3	4	5
My dog is active as he/she has been	1	2	3	4	5

**GENERAL HEALTH**					
General health compared to last evaluation	1<br>Worse	2	3<br>Same	4	5<br>Better
General health compared to initial diagnosis of cancer	1<br>Worse	2	3<br>Same	4	5<br>Better
Quality of life	Very poor ______________________________ Excelent				
